# Spontaneous non-aneurysmal subarachnoid hemorrhage in Takayasu arteritis: a case implicating hyperperfusion and cerebral dysautoregulation

**DOI:** 10.1259/bjrcr.20180113

**Published:** 2019-02-01

**Authors:** Hena Joshi, Jason Allen, Deqiang Qiu, Junjie Wu, Fadi Nahab, Karen Law, Ranliang Hu

**Affiliations:** 1 Department of Radiology and Imaging Sciences, Emory University, Atlanta, Georgia; 2 Department of Neurology, Emory University, Atlanta, Georgia; 3 Department of Medicine, Emory University, Atlanta, Georgia

## Abstract

Takayasu arteritis (TA) is a systemic chronic inflammatory large-vessel vasculitis that affects the aorta, its major branches, and the pulmonary arteries. In this report, we describe a case of a young female with TA presenting with spontaneous subarachnoid hemorrhage (SAH), an unusual manifestation of the disease. Magnetic resonance angiography (MRA) of the head and neck demonstrates multifocal carotid and vertebral arterial stenoses, but no aneurysm or vascular malformation to account for SAH. A novel and unexpected finding in this case was increased cerebral perfusion in the right frontotemporal parenchyma and transient abnormally reduced augmentation of flow in response to the cerebral vasodilator acetazolamide. The etiology of SAH thus may be related to hyperperfusion and loss of cerebrovascular autoregulation leading to small vessel damage.

## Case presentation

A 32-year-old female presented to the emergency department with transient left-sided weakness and headache. She had been diagnosed with Takayasu arteritis 1 year prior to this episode, but otherwise had no other risk factors for cerebrovascular disease. Her physical examination was normal. Laboratory tests were unremarkable except for elevated serum inflammatory markers including C-reactive protein of 39.4 mg l^−1^ (normal <10 mg l^−1^) and erythrocyte sedimentation rate of 81 mm/hr (normal <25 mm/hr).

## Imaging findings

**Figure 1.**
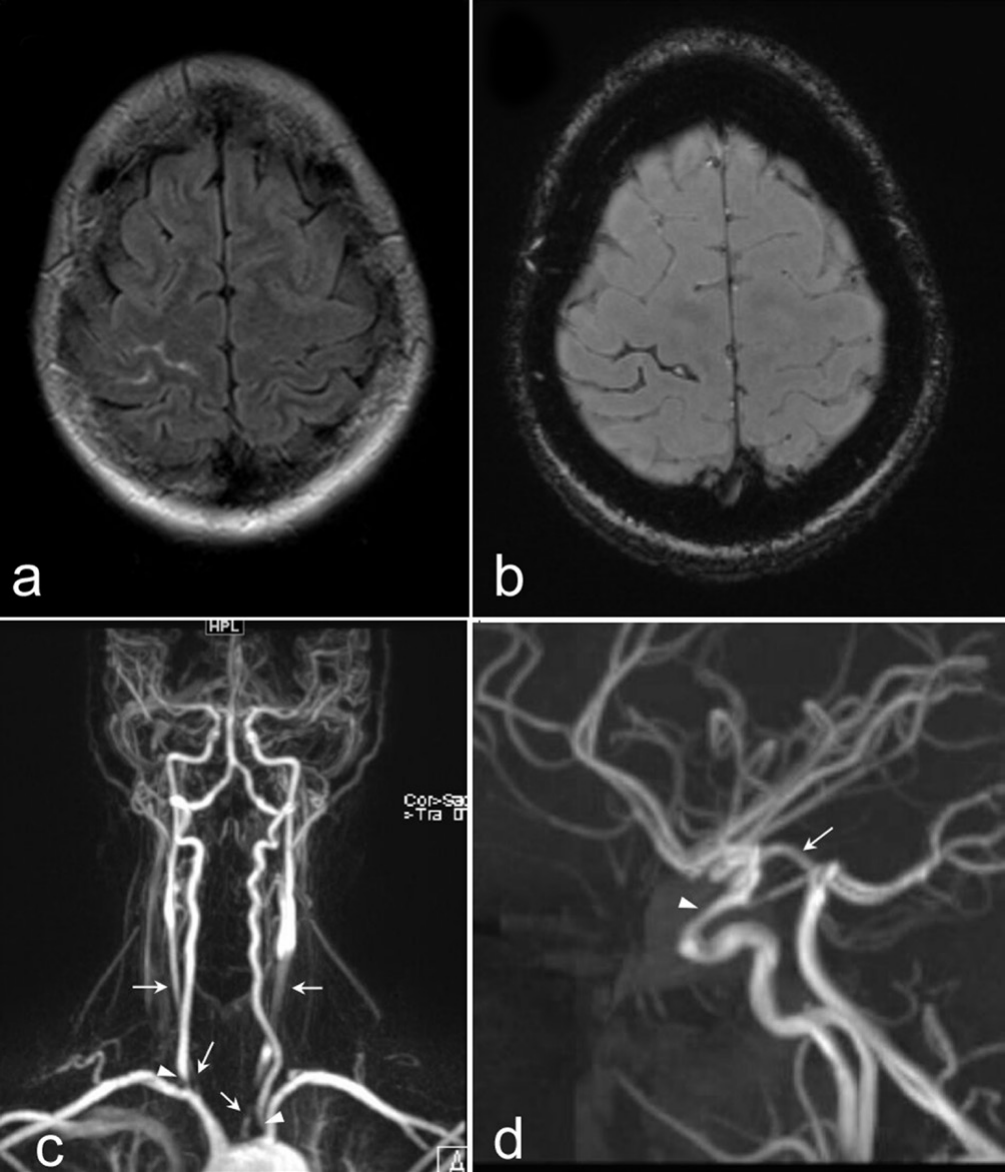
(a) Axial T2 FLAIR MRI of the brain and (b) corresponding SWI demonstrating small volume right frontal lobe subarachnoid hemorrhage (c) Coronal MIP of MRA of the head and neck showing severe stenoses of the bilateral common carotid arteries (arrows) and at the origins of the vertebral arteries (arrowheads) (d) Sagittal MIP of brain MRA demonstrates narrowing of the cavernous and supraclinoid segments (arrowhead) of the right internal carotid artery with prominent right posterior communicating artery (arrow), which may explain altered vascular supply and chronic frontotemporal hyperperfusion. No aneurysm was identified. FLAIR, fluid-attenuated inversion-recovery; MIP, maximum intensity projection; SWI, susceptibility weighted imaging.

MRI of the brain demonstrated small volume subarachnoid hemorrhage over the right frontal convexity (Figure 1a) but was otherwise normal, including negative for acute ischemia given normal diffusion-weighted imaging and apparent diffusion coefficient. On magnetic resonance angiography (MRA) of the head, there was severe stenosis of the cavernous and supraclinoid segments of the right internal carotid artery. MRA of the neck demonstrated severe stenosis at the origin of the right vertebral artery, long segment severe stenosis of the proximal to mid right common carotid artery, and multifocal severe stenoses of the left common carotid artery (Figure 1b). Mild narrowing of the bilateral proximal common carotid arteries and the subclavian arteries were also present. No intracranial aneurysm or other vascular malformation was identified.

**Figure 2.**
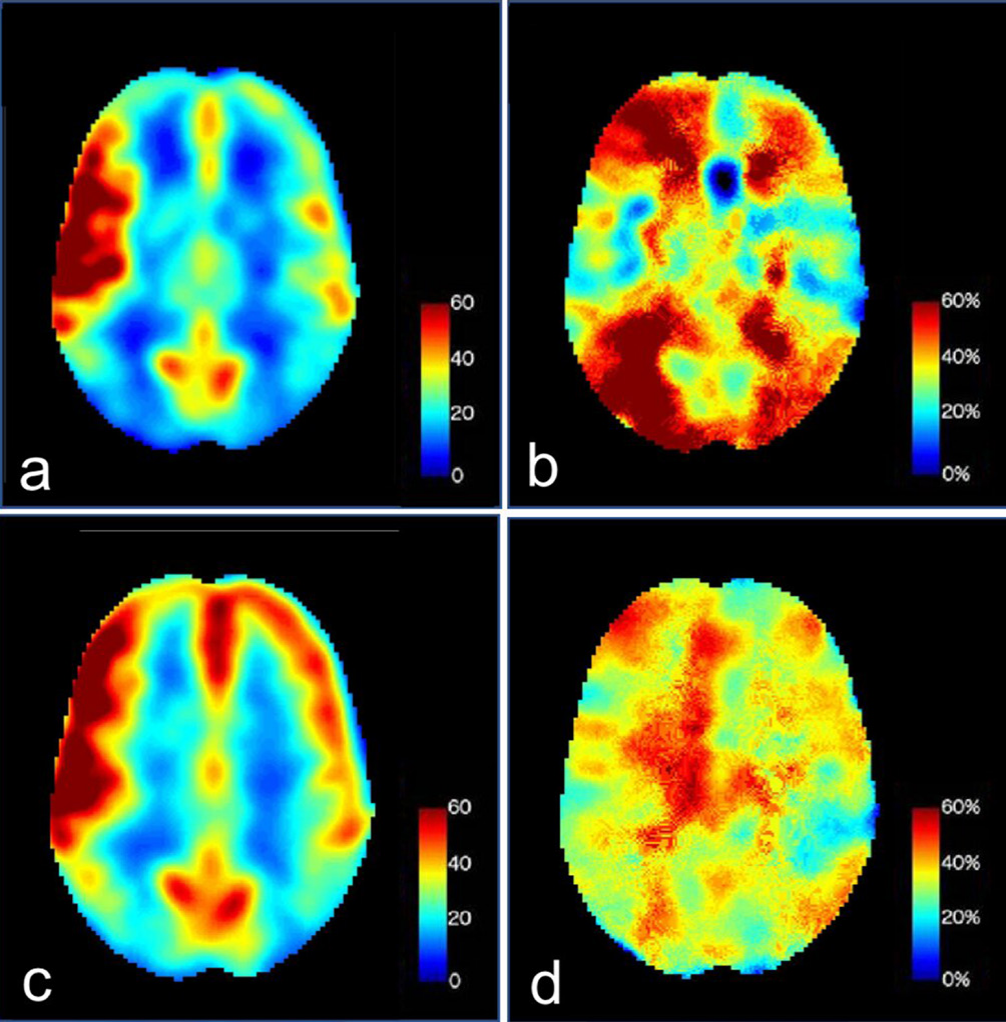
(a) ASL perfusion imaging demonstrating increased right frontotemporal cerebral blood flow and (b) cerebral vascular reactivity ASL perfusion imaging after acetazolamide administration showing relatively reduced augmentation of flow in the same region. There is robust augmentation in the remaining right cerebral hemisphere while the left cerebral hemisphere augments normally. On follow-up examination, there was (c) persistently elevated cerebral blood flow in the right frontotemporal region and (d) normal bilateral augmentation of flow during acetazolamide challenge. ASL, arterial spin labeling.

Evaluation of cerebral perfusion with three-dimensional arterial spin labeling (ASL) and acetazolamide challenge was performed. The pre-acetazolamide images demonstrated increased cerebral perfusion in the right frontotemporal parenchyma (Figure 2a). After the administration of acetazolamide, there was decreased augmentation of flow in this region but robust augmentation of blood flow in the remaining right cerebral hemisphere (Figure 2b). The left cerebral hemisphere had relatively less augmentation compared to the right but remained within normal range.

## Outcome/Follow-up

After treatment with high-dose steroids and immunosuppressive therapy, the patient was discharged from the hospital in stable condition. A follow-up MRI obtained 3 months after the initial study demonstrated resolution of subarachnoid hemorrhage and no new abnormalities. There was persistently increased perfusion in the right frontotemporal parenchyma with normal augmentation of flow on acetazolamide challenge (Figure 2c,d). Follow-up MRA of the head and neck obtained at 5 months demonstrated persistent multifocal arterial stenoses that were unchanged.

## Discussion

Takayasu arteritis (TA), also referred to as pulseless disease, is a rare chronic large vessel vasculitis that involves the aorta, its branch vessels, and the pulmonary arteries.^1,2^ It is seen more frequently in younger females and Asian populations.^2^ Arterial inflammation ultimately leads to multiple stenoses, occlusions, and aneurysms.^1,2^ Clinical manifestations of the disease depend on the phase. In the early “pre-pulseless” systemic phase, non-specific symptoms such as fever, malaise, weight loss, arthralgias, and myalgias are present.^1,2^ The chronic phase of TA presents with symptoms of end-organ ischemia such as angina, claudication, syncope, and neurological impairment.^1,2^ Physical examination may demonstrate diminished or absent pulses and vascular bruits. Diagnosis of TA is made with a combination of clinical findings, laboratory testing, and imaging findings. High-dose corticosteroids are the first-line treatment of TA with the goal of reducing inflammation and maintaining vascular competency.^2^ Interventional or surgical revascularization may be performed for symptomatic stenoses or occlusions.^2^ Spontaneous subarachnoid hemorrhage (SAH) in the absence of intracranial aneurysm such as seen in this case is an unusual manifestation of TA, and there are only three case reports describing this association in literature.^3–5^ The mechanism of hemorrhage is unclear and is presumed to be the result of hemorrhage secondary to vascular inflammation related to TA or vascular dysregulation.

Imaging features of TA include long, smooth, and tapered arterial stenosis on angiography.^2^ Cross-sectional imaging may demonstrate arterial wall thickening.^2^ To evaluate cerebral perfusion, we used arterial spin labeling, an MRI technique that magnetically labels inflowing arterial blood to measure perfusion and does not require the use of contrast agent.^6^ ASL has been used to study perfusion alterations in the imaging of dementia, stroke, and neoplasms. This technique can also be used to measure cerebrovascular reserve with acetazolamide, a carbonic anhydrase inhibitor that induces a 20–30% increase in cerebral blood flow in healthy individuals.^6^ When cerebral vasculature is maximally dilated at baseline, such as in flow-limiting cerebrovascular disease, there is lack of flow augmentation and possibly decreased flow in response to acetazolamide.^6^

An unusual finding in our case was hyperperfusion in the right frontotemporal parenchyma and reduced augmentation of flow in this region after administration of acetazolamide, whereas the surrounding tissues demonstrated robust blood flow augmentation. The etiology of increased perfusion was unclear and initially thought to be due to either luxury reperfusion following ischemia, increased blood flow due to active inflammation, or subclinical seizure activity from SAH. The follow-up examination demonstrated persistently increased perfusion in this region with normal augmentation on acetazolamide administration, thus making these transient effects unlikely. As such, chronic hyperperfusion and loss of cerebrovascular autoregulation leading to vessel damage may explain the subarachnoid hemorrhage. This is further supported by altered vascular supply in this patient due to severe bilateral carotid arterial stenoses, with the anterior circulation largely dependent on supply from the posterior communicating arteries. As her right posterior communicating artery was dominant, hyperperfusion of the right MCA territory in a chronically vasodilated state may be the cause of right frontal convexity subarachnoid hemorrhage.

## Learning points

1. Takayasu arteritis is a large vessel vasculitis that involves the aorta, its branches, and the pulmonary arteries, but can also involve the cerebrovascular system.
2. Non-aneurysmal subarachnoid hemorrhage is an unusual presentation of Takayasu arteritis with limited reported cases in literature.
3. In the present case, arterial spin labeling with acetazolamide challenge demonstrated elevated cerebral blood flow in a region with adjacent subarachnoid hemorrhage with associated, transient blood flow augmentation at presentation. This suggest that loss of cerebrovascular autoregulation and small vessel damage may be the pathophysiological mechanism of subarachnoid hemorrhage.
